# Sensorimotor supremacy: Investigating conscious and unconscious vision by masked priming

**DOI:** 10.2478/v10053-008-0029-9

**Published:** 2008-07-15

**Authors:** Ulrich Ansorge, Odmar Neumann, Stefanie I. Becker, Holger Kälberer, Holk Cruse

**Affiliations:** Department of Psychology, Universität Bielefeld, Bielefeld, Germany

**Keywords:** masked priming, vision, sensorimotor processing, attention

## Abstract

According to the sensorimotor supremacy hypothesis, conscious perception draws on motor action. In the present report, we will sketch two lines of potential development in the field of masking research based on the sensorimotor supremacy hypothesis. In the first part of the report, evidence is reviewed that masked, invisible stimuli can affect motor responses, attention shifts, and semantic processes. After the review of the corresponding evidence – so-called masked priming effects – an approach based on the sensorimotor supremacy hypothesis is detailed as to how the question of a unitary mechanism of unconscious vision can be pursued by masked priming studies. In the second part of the report, different models and theories of backward masking and masked priming are reviewed. Types of models based on the sensorimotor hypothesis are discussed that can take into account ways in which sensorimotor processes (reflected in masked priming effects) can affect conscious vision under backward masking conditions.

## Introduction

Conscious visual perception is inert; it is not instantaneous. A conscious visual percept corresponding to a specific distal and proximal visual stimulus can be altered up to about 100-250 ms after the onset of the stimulus. This has been demonstrated by visual backward masking studies (cf. Enns & Di Lollo, 2000; Stigler, 1910). In visual backward masking, a temporally trailing visual masking stimulus is presented after a visual test stimulus (for reviews see Breitmeyer, 1984; Breitmeyer & Öğmen, 2000). As a consequence of the masking stimulus, visibility of the preceding test stimulus’ features, such as its brightness, shape, or color, can be diminished or even completely prevented (Klotz & Neumann, 1999).

From an evolutionary perspective, inertia of conscious visual perception, as testified by backward masking, is puzzling. Consider the sort of problems that visual agents, such as humans, have to solve: Successful motor action (e.g., self locomotion, grasping, pursuit tracking of moving objects by the eye, etc.) requires synchronization of motor latencies with realities. Therefore, it seems that agents need to instantaneously update the flux of changing visual input in consciousness for conscious vision to catch up with the real world (cf. Nijhawan, 2002). From this perspective, the delay of conscious visual perception relative to the real world appears to be harmful: It adds to the agent’s motor latencies to make them lag behind the environmental conditions.

### Masked priming

 In the course of progress in masking research, however, the puzzle of inert conscious visual perception dissolved. Visibility and visual processing in the ser-vice of motor action (in the following referred to as *visual sensorimotor processes*) turned out to be different matters (Fehrer & Raab, 1962; Klotz & Neumann, 1999): Exactly those visual faculties that would suffer most from inert conscious perception – that is, visual sensorimotor processes, are spared under invisibility conditions (Bridgeman, 1992; Bridgeman, Lewis, Heit, & Nagle, 1979; Goodale & Milner, 1992; Neumann & Klotz, 1994). Fehrer and Raab (1962), for example, had their participants react to backward-masked visual stimuli, and found that responses to the subjectively invisible stimuli were as efficient as responses to clearly visible stimuli. 

Yet, Fehrer and Raab required a single, uniform reaction to each and every stimulus. Thus, their conclusion was doubtful for it was unclear whether participants indeed reacted to the invisible stimuli, as Fehrer and Raab thought, or whether participants were reacting to the mask, with the masked stimulus pre-warning for the upcoming mask and, thus, reducing the time necessary to (a) perceive the mask and (b) respond to it (Neumann, 1982).

It was not before the advent of the masked priming paradigm that it was demonstrated that participants can respond to an invisible stimulus shown below the threshold of conscious awareness (Marcel, 1983; Wolff, 1989). In the masked priming paradigm, the backward-masked test stimulus is not the main target of action (in contrast to investigations such as that of Fehrer & Raab). Instead, the test stimulus is an accessory stimulus that precedes the clearly visible target. The test stimulus is called “a prime” because of its facilitating (or interfering) effect on the response to the clearly visible target (Klotz & Wolff, 1995; Wolff, 1989).

**Figure 1.**
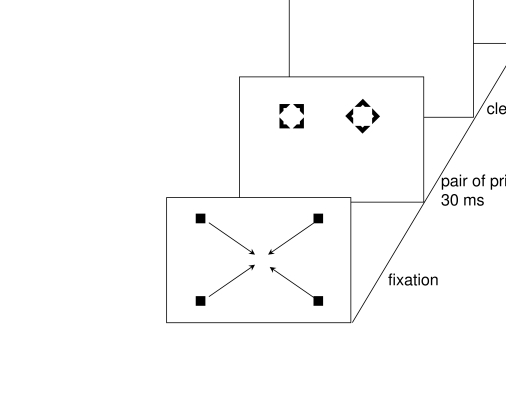
Depicted is a congruent trial, with a masked target-shaped prime (e.g., a square) on the same side as the visible target shape; procedure after Klotz and Neumann (1999). Arrows stand for motion of the fixation dots (toward the screen center). For details refer to the text.

For an example of the masked priming procedure, take a look at Figure 1, where stimuli and trial details of the study of Klotz and Neumann (1999) are depicted. In each trial of their study, Klotz and Neumann showed their participants a pair of clearly visible geometric figures, a square and a diamond, with one of the figures presented left and the other one right of fixation. These geometric figures served two purposes. First, one of the figures was the target for the responses of the participants (the other figure was a distractor): Half of the participants responded to the position of the square as a target, with a left-hand key press if the square was left and a right-hand key press if the square was right. (These participants had to ignore the diamonds as distractors.) The other half of the participants responded in a corresponding manner to the position of the diamonds (and had to ignore the squares as distractors).

The clearly visible geometric figures also served, secondly, as backward masks that prevented the visibility of a pair of preceding primes, one presented at the same position as the target and one at the position of the distractor. To mask the primes, the visible square and diamond were slightly larger than the primes such that their inner contours exactly fitted around the outer contours of the smaller primes. Thereby, ideal conditions for metacontrast masking of the primes by the larger target and distractor figures were created (cf. Breitmeyer & Öğmen, 2006).

As it can be seen in Figure 1, primes were also geometric figures, smaller than the target and the distractor but otherwise very similar to them. The crucial variation concerned whether or not the prime indicated the same response as the target. In congruent conditions, the pair of masked primes had a target-like prime shape at the position of the upcoming target shape. For example, a square-shaped prime on the left preceded a square-shaped target on the left. Hence, if it were true that an invisible (backward-masked) prime can activate a response, facilitation of the response to the target was to be expected because prime and target indicated the same response (i.e., a left-hand key press in the example). In incongruent conditions, the pair of masked primes had a target-like prime shape at the position opposite to that of the upcoming target shape. For example, a square-shaped prime on the left preceded a square-shaped target on the right. If it were true that this invisible prime can activate a response, interference with the response to the target was to be expected because prime and target indicated alternative, mutually exclusive responses (i.e., in the example the prime indicated a right-hand key press, whereas the target required a left-hand key press). Facilitation in congruent conditions and interference in incongruent conditions were expected to show up in comparison to a neutral baseline condition without a target-like shape prime. For example, if squares were used as targets, the pair of masked stimuli in the neutral condition consisted of two diamonds.

 Results of studies by Neumann and Klotz (1994), and Klotz and Neumann (1999) nicely supported these expectations. RTs (Reaction Times) under congruent conditions were shorter than under neutral conditions, and RTs under incongruent conditions were longer than under neutral conditions. (A corresponding trend was observed in the error rates.) Importantly, RT effects accomplished with the masked priming paradigm cannot be attributed to the facilitation of mask perception by the pre-warning primes because such a pre-warning would have led to equal facilitation under congruent, neutral, and incongruent conditions. 

In essence, the masked-priming procedure directly pits the effect of the invisible prime against that of the visible target: The prime indicates one specific response and the target signifies a second, frequently alternative response (Marcel, 1983; Wolff, 1989). There are now numerous studies that have confirmed that under these conditions, an invisible prime activates a motor response that can delay the response required for the visible target or that occurs instead of the response to the target (cf. Ansorge, 2003; Ansorge, Klotz, & Neumann, 1998; Breitmeyer, Ro, & Singhal, 2004; Eimer, 1999; Klotz & Neumann, 1999; Klotz & Wolff, 1995; Leuthold & Kopp, 1998; Neumann & Klotz, 1994; Schmidt, 2001, 2002; Schmidt, Niehaus, & Nagel, 2006; Vorberg, Mattler, Heinecke, Schmidt, & Schwarzbach, 2003; for a review see also Schmidt, this volume).

In addition, although results from the masked priming paradigm are maybe the most unequivocal evidence in favor of spared visual sensorimotor processing capacities under invisibility conditions, they are by far not the only evidence. Goodale, Milner, Jakobson, & Carey (1991), for instance, observed that for an agnostic patient (DF), her object agnosia rendered visual size and orientation information invisible and yet the patient was able to use the very same visual information successfully in the sensorimotor domain, for purposes such as grasping and wrist rotation.

### The sensorimotor supremacy hypothesis

According to the sensorimotor supremacy hypothesis, dissociability between visual sensorimotor processing and conscious visual perception is due to different functions and onsets of the respective processing mechanisms during the course of phylogenetic evolution and ontogenetic development. To start with, sensorimotor visual processing is an achievement meeting a pressing and pertinent problem in human evolution and ontogenetic development because visual sensorimotor processing is necessary for successful coordination of behavior within the visual environment: Numerous life-maintaining behaviors, such as feeding, procreation, etc., draw on the human’s capacity to use visual information to anticipate and to control its grasping movements, gait, eye movements, and so on.

By contrast, conscious visual perception does not solve a similarly pressing problem in the course of evolution or ontogenetic development. Conscious visual processing more likely serves purposes such as maintaining a visual image beyond its physical duration (cf. Hardcastle, 1995; Neisser, 1967), thus making it available for more diverse purposes after its initial representation (cf. Dehaene & Naccache, 2001). Therefore, according to the sensorimotor supremacy hypothesis, visual sensorimotor processing is the more fundamental and ancient adaptation in comparison to conscious vision.

In line with this assumption of the sensorimotor supremacy hypothesis, conscious visual perception as the more recent evolutionary achievement seemingly builds on the more ancient visual sensorimotor processing capacities (cf. Helmholtz, 1879; O’Regan & Noë, 2001). Slightly moving the eyes, for instance, is a necessary prerequisite for conscious visual perception: Stabilization of the retinal image by moving the image in accord with the eyes is known to rapidly lead to a fading of the conscious visual percept (Riggs, Ratliff, Cornsweet, & Cornsweet, 1953). In general, according to the views that are summarized as the sensorimotor supremacy hypothesis, visual sensory input in a first step provides a prediction that is secondly validated by comparing it with predicted premotor or motor consequences, with thirdly conscious visual perception corresponding to only the confirmed predictions. On a phenomenal level, the standard everyday experience accompanying this processing cycle is that of visual gist perception (cf. Neisser, 1967) being transformed into a conscious visual percept segregated into center and fringe (cf. James, 1890). On the motor and premotor level, eye movements, such as saccades (e.g., Wolff, 2004), and visuospatial attention shifts preceding the saccades (or even occurring instead of them) (cf. Neisser, 1967; Simons & Rensink, 2005; Treisman & Gelade, 1980) are most frequently used for the purpose of conscious visual perception. Detection of change across images, for example, depends on a prior shifting of visuospatial attention to that position in the image plane at which the change occurs (Simons & Rensink, 2005). Therefore, conscious visual perception comes at the price of a delayed latency, with a less than perfect temporal resolution, because the correlations which give rise to conscious visual perception can only be derived after the sensory inputs *and* their temporally trailing motor or premotor consequences. Yet, as will be discussed in the next passage, this proposed role of sensorimotor processing for conscious visual perception seems to be in conflict with a long-standing notion that we will refer to as the *inflexibility assumption*.

### Processing unconscious visual input: flexible or inflexible?

According to the inflexibility assumption, visual faculties that are independent of consciously perceived input are inflexible, strongly automatic, or hard-wired (cf. McCormick, 1997; Posner & Snyder, 1975). This means that unconscious input presented below the threshold of awareness can only be processed in a fixed manner: The corresponding processes are inflexible or not malleable.

If the inflexibility assumption were true, the sensorimotor supremacy hypothesis, as outlined above, would seem to be faced with a paradox. On the one hand, the sensorimotor supremacy hypothesis claims that visual sensorimotor processes have to precede conscious visual perception to fulfill their validating function for conscious perception: Premotor or motor consequences need to be correlated with their preceding sensory inputs before conscious visual perception of these inputs. On the other hand, the inflexibility of the processing of a particular unconscious visual input stimulus seems to severely limit (a) the range of possible motor effects that can be used as a correlating consequence of that input, and thus also (b) the range of possible correlations between input and output.

With respect to these concerns, however, it should be noted that two sorts of inflexibility have to be discerned that create the seeming paradox only if they are confused with one another. First, inflexibility or non-malleability of *input-output relations* means that each and every particular visual input can only lead to a limited class of particular motor outputs as valid transformations of the input, once a particular action is intended. Let’s say that we intend to point in the direction of a light, positioned 45° to the left of our straight-ahead viewing direction. Under these conditions, a valid transformation of the input would be a pointing direction that is at least approximately in the direction 45° to the left, and certainly pointing 45° to the right would be a violation of the intended motor output.

This sort of input-output inflexibility is undisputed. In fact, it is necessary for the functional role of sensorimotor processing in validating conscious visual perception. If one and the same visual input could have any of several motor effects as its valid output, the motor output could not be predicted, and correlating input and output would not be used to confirm the content of conscious visual perception. Thus, according to the sensorimotor supremacy hypothesis, once a particular motor output has been intended, input-output transformations should indeed proceed in a fixed manner.

However, this sort of input-output relation inflexibility must be carefully discerned from a second sort of inflexibility: *output selection inflexibility*. Output selection inflexibility means that the unconscious stimulus input determines which motor actions can be performed. Thus, output selection inflexibility means that an agent cannot *intentionally* decide in advance of the stimuli about the way that she or he wants to use the visual input for her or his motor action. This second sort of inflexibility is sometimes considered as being characteristic of processing unconscious visual stimuli (cf. McCormick, 1997). From the standpoint of the sensorimotor supremacy hypothesis, however, output selection inflexibility is clearly denied. Even more important, output selection inflexibility is not supported by the empirical facts.

According to the best known theory defending this particular assumption of the sensorimotor supremacy hypothesis, the so-called *direct parameter specification (DPS) account* (Neumann, 1989, 1990; for related conceptions see Kiefer, this volume; Kiesel, Kunde, & Hoffmann, this volume), it is possible to intentionally choose, in advance of an unconscious visual input, among several different motor effectors (e.g., eyes vs. hands) and among several different motor parameters (e.g., direction vs. distance of to-be-grasped objects) as potential motor variables to be specified by unconscious visual input. To stay with our example, with an input consisting of a visual light positioned 45° to the left, DPS predicts that we can also successfully intend to point in a direction 90° shifted to the right of the visual input light. Once we have prepared such an action plan in advance of the input, an unconscious light positioned 45° to the left should lead to a pointing response 45° to the right of the straight-ahead viewing direction as its predicted and valid motor consequence. (Note that input-output inflexibility would still be necessary to tell valid from invalid responses.)

 In line with this assumption of intentional flexibi-lity in choosing among different motor outputs, visual agents are able to flexibly tailor the consciousness-dissociated visual sensorimotor processing to their currently intended actions (Ansorge, 2004; Ansorge, Heumann, & Scharlau, 2002; Ansorge & Neumann, 2001, 2005; Eckstein & Perrig, 2007; Kunde, Kiesel, & Hoffmann, 2003; Leuthold & Kopp, 1998; Neumann & Klotz, 1994; Reynvoet, Gevers, & Caessens, 2005; Schlaghecken & Eimer, 2004). Neumann and Klotz (1994), for instance, found that participants were able to intend different actions as instructed under different conditions and thus were able to use one and the same unconscious visual stimulus input equally well for the purpose of different motor responses. Specifically, participants were able to either respond in the direction of an unconscious visual input stimulus (e.g., they activated a left-hand key-press in response to a left unconscious stimulus) or in the direction opposite to that input (e.g., they activated a right-hand key-press in response to a left unconscious stimulus). 

### Summary

To summarize, the sensorimotor supremacy hypothesis assumes that visual sensorimotor processing temporally precedes conscious visual perception. Phylogenetic and ontogenetic progression is from the more basic building blocks of visual sensorimotor processing – meeting the most pressing demands – to the more advanced levels of conscious vision. Conscious visual perception, by contrast, is based on a comparison of intended motor outcomes with what has actually been done (Cruse, 2003; Helmholtz, 1879; Hoffmann, 1993). In line with that assumption, visual sensorimotor processing is dissociable from visual conscious perception (cf. Klotz & Neumann, 1999).

### Open questions

Despite the above summarized progress in our understanding of the interplay between conscious and unconscious visual processes, several obstacles remain for a unified theory of masked priming or unconscious vision. In the following, we identify two of them and propose ways how the principle of sensorimotor supremacy could be used to understand and empirically approach the outstanding questions.

First, we will review evidence from visual backward-masking studies concerned with the shifting of visuospatial attention toward masked invisible stimuli and with the semantic processing of masked invisible stimuli. Obviously, faculties of unconscious vision reflected in masked motor priming could be different from those responsible for masked attentional priming and masked semantic priming effects. However, one part of the sensorimotor supremacy hypothesis we intend to put forward is that all of the different masked priming effects could be explained by a unified principle of consciousness-dissociated visual sensorimotor processing. We will end this first passage with a sketch of empirical means suited to test a unified account of masked priming effects based on the principle of sensorimotor supremacy.

In the second part, we will briefly review current theories that account for backward masking. From this review, we conclude that masking theories have so far not fully incorporated the potential implications of findings from masked priming studies. In particular, with few exceptions, existing masking theories treat sensorimotor priming as inconsequential for what is consciously seen under masking conditions. By contrast, we will outline a type of sensorimotor supremacy model that regards priming effects as being causally responsible for what is perceived under masking conditions. The model draws on the established attentional effect that a masked prime exerts on the conscious visual perception of the mask (cf. Neumann, 1982), and extends the explanation to the sensorimotor level. We will conclude the second review with a brief outline of new testable predictions derived from a sensorimotor supremacy model of visual masking.

## Testing a unified account of masked priming effects

### Masked priming: motor, attentional, and semantic

A visually (backward) masked, and thus invisible, prime can have at least three different effects. In terms of the procedures that have been used, these effects are definitely different from one another. However, whether or to what extent there also exist similarities between the effects, and whether it is necessary to give different accounts for these effects, is debatable under the perspective of the sensorimotor supremacy hypothesis. This will be detailed in the following paragraphs.

First, it is relatively certain that under appropriate conditions, an invisible prime can activate a motor response. Neumann and Klotz (1994), for example, used a pair of black bars as a clearly visible target, and asked their participants to respond to that target’s position. With a target on the right, observers had to press a right-hand key, and with a target on the left, they had to press a left-hand key. Prior to the target, a pair of masked smaller black bars was presented as a prime. Under these conditions, the prime facilitated the response if it was presented at the target’s position, and it interfered with the response if it was presented at a position away from the target. Interference and facilitation were evident in comparison to a neutral baseline condition without a masked prime (see also Klotz & Neumann, 1999, and above).

Several lines of evidence corroborated the conclusion that this priming effect reflected sensorimotor processes. Leuthold and Kopp (1998) used the procedure of Neumann and Klotz, and showed that prime-induced interference was also reflected in the direction of the lateralized readiness potential of the EEG, a known correlate of pre-motor and motor activation, mostly originating in the primary motor cortex (Leuthold & Jentzsch, 2002). Similar results were found by using slightly different procedures (Dehaene et al., 1998; Eimer & Schlaghecken, 1998).

Another approach was made by Neumann and Klotz (1994), and Ansorge and Neumann (2005). These authors noted that the two factors of (a) similarity/dissimilarity of responses activated by prime and target, respectively, and of (b) sensory similarity/dissimilarity between prime and target, were confounded in masked priming studies. Therefore, they wanted to rule out that masked priming effects were merely due to sensory processes, that is, to the lower sensory similarity between prime and target in interfering relative to facilitating conditions. To that end, they used the same sensory conditions in both sensorimotor interfering and sensorimotor facilitating conditions: The masked prime was always presented with the same distance and at the same position away from the target, but the prime required the same response as the spatially distant target in some conditions, whereas it required a response other than the target in alternative conditions. Again, in line with a sensorimotor interpretation (i.e., a response activation effect), and disproving an account merely in terms of sensory prime-target similarity, interference by the prime was observed if the prime indicated a response other than the target relative to a condition where the prime signified the same response as the target.

Still another line of evidence was provided by Vorberg et al. (2003). These authors asked their participants to respond in the direction of a visible target, either a left or a right pointing arrow. As a prime, they used a backward-masked target-preceding (smaller) arrow. The prime either pointed in the same direction as the target or in the opposite direction. The prime-target interval varied from a single refresh of the computer screen to about 100 ms. The most important observation of Vorberg et al. (2003) was that, with an interfering invisible prime, error probability was a function of the prime-target interval. The probability of an erroneous response in the direction of an interfering prime arrow (pointing in the opposite direction to the target) increased with the time by which the prime arrow was presented before the visible target arrow. This finding is in line with a motor activation effect: The prime is able to activate a response corresponding to its direction. This motor activation eventually leads to an overt response if it is not sufficiently quickly countermanded by a competing response activated by the visible target.

A second kind of masked priming effect is of an attentional origin. According to a widely held notion, the abrupt onset of a visual stimulus in the periphery of the visual field captures attention automatically (cf. Jonides, 1981; Yantis & Jonides, 1984), at least if the features of the visual stimulus are sufficiently task-relevant (cf. Ansorge & Heumann, 2003, 2004; Folk & Remington, 1998, 1999; Folk, Remington, & Johnston, 1992). Along these lines, Neumann (1982; see also Neumann & Scharlau, in press) argued that a backward-masked, invisible prime presented prior to a visible mask and at the mask’s position should facilitate the visual perception of the mask, under the following two assumptions: (a) visual conscious perception of a stimulus depends on a prior shifting of attention to (or focusing of attention on) the position of the perceived stimulus (cf. Neisser, 1967; Simons & Rensink, 2005), and (b) an invisible stimulus (such as a backward-masked prime) is capable of capturing visuospatial attention (cf. McCormick, 1997). If both assumptions hold true, an invisible prime preceding a visible mask at its position should attract attention. As a consequence, attention would be already at the position of the mask when the mask has its onset. Thus, the prime should shorten the delay until the mask can be consciously perceived (cf. Neumann, 1982).

A very similar prediction can be made on the basis of the perceptual retouch theory (Bachmann, 1984, 1994). According to the perceptual retouch theory, conscious perception of a visual stimulus requires two steps, (a) an initial onset response evoked in the visual cortex, and (b) a second signal that confirms this initial cortex response, with the confirmation signal being delayed by about 80 ms relative to the initial brain response. According to the perceptual retouch theory, the masked prime evokes its corresponding initial brain response, but once the delayed confirmatory signal reaches the visual cortex, mask-induced activity prevails at the location formerly occupied by the prime and is confirmed instead of the already passed prime-induced activity. Thus, according to the perceptual retouch theory too, a backward-masked prime should shorten the time to consciously perceive a visual mask (but see Scharlau, Ansorge, & Horstmann, 2006, for differences between the predictions of perceptual retouch theory and an explanation by visuospatial attention).

In several investigations, Scharlau and her colleagues bore out the attentional hypothesis (e.g., Scharlau, 2002, this volume; Scharlau & Neumann, 2003a, 2003b). Scharlau’s general procedure requires participants to give a temporal order judgment (TOJ) about which of two visible stimuli comes first, with the interval between these two stimuli varying from concomitant onsets to some tens of milliseconds between their respective onsets. If a masked prime is presented as a third stimulus in advance and at the position of only one of the other two stimuli, the primed stimulus of the two latter stimuli seems to temporally lead the unprimed stimulus even under conditions where both primed and unprimed stimulus have had a concomitant onset.

Further support for an attentional effect of the masked prime was provided by Jaśkowski and colleagues (Jaśkowski, Skalska, & Veleger, 2003). These authors used a negative event-related potential at stimulus-contralateral, posterior scalp sites to track where participants directed their visuospatial attention, and were able to demonstrate that attention was directed toward a backward-masked invisible prime (see also Ansorge & Heumann, 2006). Very similar results have been obtained with stimuli that were backward-masked by four dots (cf. Woodman & Luck, 2003). So much for an attentional effect of an unconscious, masked prime.

Still, in a third variant, priming is by masked words. This has been sometimes attributed to processing within semantic memory (cf. Dehaene et al., 1998; Kiefer & Spitzer, 2000; Marcel, 1983). In the so-called masked semantic priming studies, words are used as masked primes and/or visible targets. A masked priming word which is semantically associated with an upcoming visible target word facilitates the response to the visible target word relative to a masked priming word which is not or less semantically associated with the visible target word (e.g., Cheesman & Merikle, 1985; Greenwald, Draine, & Abrams, 1996). It is commonly assumed that semantic priming by masked priming words reflects spreading mutual activation of representations of priming word and target word within an interconnected memory network. In semantic memory (or mental lexicon if one wishes to restrict the account to visual words), connections between related representations are stronger (or put another way: more facilitative) than connections between less or unrelated representations (which are also sometimes assumed to be inhibitory) (cf. McClelland & Rumelhart, 1981; Morton, 1969; Neely, 1991). As a consequence of this general architecture, the semantic representation of a masked prime can pre-activate the representation of a semantically related visual target word presented after the prime, so that a critical threshold activation value of the target-word representation that allows the recognition or discrimination of the target word is rapidly achieved.

Admittedly, many masked priming effects that were attributed to spreading activation within semantic memory can be explained equally well by sensorimotor processes. Marcel (1983), for example, asked his participants to name the color of a clearly visible target patch. Hence, a masked color word prime that denoted the color of the upcoming patch (e.g., the masked word “red” preceding a clearly visible, to be named red color patch) might have activated the correct naming response, whereas a masked color word prime that denoted a color different from that of the upcoming target patch (e.g., the masked word “green” preceding a clearly visible, to be named red color patch) could have interfered with the correct naming response.

However, in line with the spreading-activation account, a masked word priming effect is also observed where a response activation effect can be ruled out. Kiefer (2002), for instance, used a lexical decision task: In each trial, a word or a nonword was presented as a visible target, and participants had to decide whether the target was or was not a word. Therefore, the priming word always indicated the same response (i.e., a ‘word’ response). Yet event-related potentials were affected by the amount of semantic association that existed between the masked priming word and visible target word: Less semantically target-associated masked priming words induced a stronger N400 – a component reflecting semantic language processes (cf. Kutas & Hillyard, 1980) – than more target-associated masked priming words.

Different time courses of priming effects with (a) masked words vs. (b) masked-shape/location stimuli lent additional indirect support for the distinction between, on the one hand, semantic priming and on the other hand, sensorimotor priming as reflecting distinctive processes. Research with masked location or shape primes showed that sensorimotor effects of the masked prime reverse with a prime-target interval beyond about 100 ms (cf. Eimer & Schlaghecken, 1998; Jaśkowski & Verleger, this volume; Schlaghecken, Rowley, Sembi, Simons, & Whitcomb, this volume; Sumner, this volume), at least if the masked prime is both task-relevant and similar to one of the visible targets (Eimer & Schlaghecken, 1998, 2001; Klapp & Hinkley, 2002; Lleras & Enns, 2004, 2005, 2006; Schlaghecken & Eimer, 2002, 2004; Verleger, Jaśkowski, Aydemir, van der Lubbe, & Groen, 2004). By contrast, the priming effect of masked word primes typically does not invert with an increasing prime-target interval. It follows a different time course, being present with relatively short prime-target intervals (< 100 ms) but absent with longer prime-target intervals (Kiefer & Spitzer, 2000). This latter finding fits well with the assumption that masked semantic priming is due to spreading activation within semantic (lexical) memory, giving way over time to slower, more deliberate processing (cf. Neely, 1977).

### A unitary account of masked priming effects?

From the review above, it should be clear that in a trivial sense, masked priming effects rely at least to some extent on different specific stimulus properties. Think of the participants discriminating between leftward and rightward pointing masked arrows (Vorberg et al., 2003). If such discrimination were not possible with masked arrows, different masked arrows should have had the same effect, which is not the case. Likewise, if participants were unable to discriminate between different electromagnetic frequencies or wavelengths (i.e., “colors”) of masked visual stimuli (cf. Breitmeyer et al., 2004; Schmidt, 2002; Schmidt et al., 2006), masked priming effects of red and black stimuli should have been the same, irrespective of whether searched-for visible targets were black or red, again a prediction which is at variance with observations (Ansorge & Neumann, 2005).

As a plausible starting point, we therefore concede that processing of masked visual stimuli might rely on different underlying mechanisms in the extent that the masked stimuli have different discriminated visual features (different colors, shapes, locations). Yet this does not preclude the possibility that different underlying processes of unconscious vision or masked priming also share important characteristics. In fact, the latter assumption is likely in light of the high similarity between different masked-priming procedures.

In the following, we will take a two-step approach to devise a test for the hypothesis of a unitary mechanism reflected in different masked priming effects. First, we outline which kind of commonality exists between different faculties of unconscious vision, starting with a discussion of sensorimotor and attentional processes, and proceeding to a theory involving also semantic processes. We will then in a second step sketch the general empirical approach that can be used to investigate whether these theoretically conceivable commonalities indeed exist.

Starting at the theoretical level, from the perspective of the sensorimotor supremacy hypothesis, all unconscious vision is of the form of sensorimotor processing. If this holds true, how can we account for masked attentional priming?

This task is easily accomplished because selectivity reflected in visuospatial attention basically serves sensorimotor control as assumed above and as we will explain in a minute. To start with, from the viewpoint of the agent, the amount of effector systems that are available to perform in a given task or situation is always restricted. For instance, humans have only two hands to grasp. The range of possible actions is even further restricted to those which can be made from the actually held effector positions in space. This limited space of possible motor actions imposes the need for selectivity reflected in phenomena of visuospatial attention (e.g., Allport, 1987; Neumann, 1987), the most pertinent example of this generalization being that visuospatial attention is used in the control of eye movements (e.g., saccades). To successfully program the direction and the amplitude of a saccade toward a visual target, a viewer has to select and incorporate sensory information about the target’s location relative to the currently fixated position. According to the premotor theory of attention, this function is served by visuospatial attention (Rizzolatti, Riggio, Dascola, & Umiltà, 1987; Rizzolatti, Riggio, & Sheliga, 1994). In line with the premotor theory, an overt saccade (as well as a pointing movement toward a visual target) is preceded by a shift of visuospatial attention toward that target’s location (Deubel & Schneider, 1996, 2004).

In conclusion, from a theoretical perspective alone it is likely that commonalities exist between unconscious visual sensorimotor processes and unconscious shifting of visuospatial attention. The common denominator is the need to select among different sensory information for the purpose of action. In other words, shifting of visuospatial attention is but a very frequently used mechanism of steering motor actions, such as saccades.

But how does semantic processing fit into the picture? According to one widely held notion, which is rooted in the initial research agenda of cognitive science, semantic information is represented in a relatively abstract or amodal manner (Newell & Simon, 1972; Pylyshyn, 1984). This means that we can disregard the hardware-dependent sensorimotor-processing level of the representing system for the analysis of its computational mnemonic functions. It is this basic contention that made it possible to simulate and study human memory by analogy to the computer. In

the extent that semantic processes occur independently of sensorimotor processes, unconscious sensorimotor processes cannot account for masked semantic priming.

However, views on semantic representations in general have changed since then. The embodied cognition view assumes that we cannot abstain from taking into account what goes on at the more basic sensory and sensorimotor level of processing if we seek to explain and to understand semantic memory processes (Barsalou, 1999; Wilson, 2002). According to Barsalou’s (1999) Perceptual Symbol Systems theory, for example, sensory and sensorimotor representations are stored as part of an original experience, and a semantic memory representation is instantiated as drawing on the representative and characteristic aspects of several of the more basic sensory and sensorimotor memory representations. From this theoretical perspective, semantic meaning and sensory/perceptual features are processed and stored by shared mechanisms, allowing for relatively similar effects and direct interactions between semantic and sensory or sensorimotor processes.

In line with the embodied cognition view, Proctor and Vu (2002), for example, found evidence for both predictions. Participants had to respond to the color of the words ‘left’ and ‘right’: Participants that had to press a left key in response to a green word, also had to press a right key to a red word. Spatial semantic meaning of the target word (i.e., its respective spatial connotation) was task-irrelevant. Under these conditions, spatial meaning of the target word nonetheless significantly affected response efficiency. A target word with a spatial meaning corresponding to the direction of the required response (e.g., the red word “right” requiring a right-key press) led to faster responses than a target word with a spatial meaning not corresponding to the direction of the response (e.g., the green word “right” requiring a left-key press).

Such results indicate that semantic representations can directly impact on sensorimotor processes, as would be predicted by the embodied cognition view: This impact is reflected in the efficiency of response execution. Moreover, similar spatial correspondence effects are observed with non-word stimuli, which have no spatial semantic meaning but which are presented either at a corresponding observer-relative location (a red stimulus on the right requiring a right key press) or at a non-corresponding position (a green stimulus on the right requiring a left key press; for a review of the effect, see Lu & Proctor, 1995). To conclude, results such as Proctor and Vu’s (2002) indicate that semantic processes and sensory or sensorimotor processes can directly interact with one another and can have comparable effects.

Proctor and Vu’s (2002) study is also a nice example of how the research in this area should be pursued. To test the unitary sensorimotor supremacy hypothesis of masked semantic, attentional, and sensorimotor priming we should look for (a) similarities between the respective masked priming effects in motor priming and semantic priming studies and (b) direct interactions between different levels of processing, such as masked semantic and masked sensorimotor priming effects, as these are reflected in task performance. Concerning the attentional effect of the masked prime, for instance, we propose to take one of the characteristics of the masked priming sensorimotor effect and to test whether it can be replicated in the attentional domain.

As an example of that kind of research, Ansorge (2003) compared temporal-nasal visual hemifield asymmetries of the strength of the masked priming effect under two conditions. In one condition, only response activation could have contributed to the masked priming effect. In another condition, visuospatial attention contributed to the masked priming effect too. Results showed that this difference between the conditions did not matter. In line with the hypothesis of a common origin of different masked priming effects, temporally presented masked primes always led to stronger priming effects than nasally presented masked primes.

**Figure 2.**
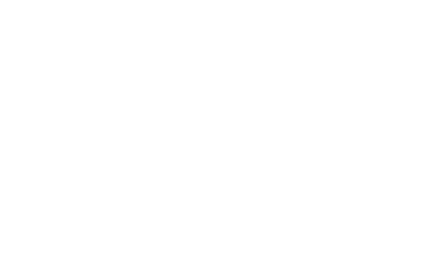
Depicted is an invalid trial, with a masked shape-singleton prime in the upper left corner (the one red diamond among the three different, shape-homogenous square primes) in the first display (depicted in the background) followed by square-shaped masks at all four positions (depicted in the foreground), with one of the masking squares serving as a target. In the depicted example trial, participants have to search for a black target square (in the upper right corner of the depicted display), and have to respond to its location (i.e., whether it is left or right). Thus, the trial is invalid because the masked singleton prime is presented at a position away from the target. Note that the masked shape-singleton prime is task-irrelevant in almost every respect. It has a color and a shape different from that of the target. Thus, participants have no reason to intentionally search for the shape or for the color of the shape-singleton prime. Furthermore, under the depicted conditions, participants have also no incentive to search for a singleton by intention, because the target is not a singleton either (neither with respect to its shape nor its color). Would the masked shape-singleton prime still capture attention away from the target? (Under the depicted conditions this prediction is made by theories assuming that attention is captured to locations containing the largest feature differences relative to the rest of the display.) The corresponding attentional effect would be reflected in posterior ERP laterality indices (compared to conditions with masked shape-singleton prime and target being presented on the same side, e.g., both being on the right). Note that under the depicted conditions, stimulus intensity in the priming display is the same at all positions. Therefore, any index of attentional capture by the masked shape-singleton prime cannot be attributed to stimulus intensity. (Arrow: direction of time.)

However, the evidence is not always in favor of a common unitary account of masked priming effects. Ansorge and Heumann (2006), for example, tried to replicate the well-established top-down contingency of the response-activation effect of masked primes (cf. Ansorge & Neumann, 2001; Kunde et al., 2003) for the attentional effect of masked primes. Using an ERP-measure of visuospatial attention, this replication failed. Yet, the results were preliminary, because in Ansorge and Heumann’s 2006 study, ERP-measures indicative of visuospatial attention were possibly contaminated by confounding sensory differences between the conditions. In particular, stimulus intensity at the position of the masked prime was greater than stimulus intensity at other positions in the display, because masked primes as in the study of Neumann and Klotz (1994) and Ansorge and Neumann (2005) were used. Remember that this means that a pair of masked bars was presented at only one of several possible positions. Figure 2 illustrates procedures (using stimuli adapted from Klotz & Neumann, 1999) that should be used in the future to circumvent the confounding stimulus intensity differences in the study of top-down control contingencies of attentional masked priming effects.

Whereas it is relatively easy to compare attentional and response-activation effects of masked primes, the situation changes if it comes to the comparison of semantic and sensorimotor effects. The reason for this is that sensorimotor and attentional effects can be studied by using the same kind of stimuli. Thus, any confounding stimulus differences between the conditions are prevented. By contrast, the same is not true for semantic and sensorimotor effects: It is hard to imagine, for example, what kind of response-activation effect would be an appropriate test of the association between the words “chair” and “table”.

 However, the problem can be solved in the latter case too. In an ingenious study, Dimberg, Thunberg, and Elmehed (2000), for example, showed that masked face stimuli with different affective expressions led to corresponding face muscle activations on the side of the observer. This study nicely illustrates that with appropriate procedures the sensorimotor supremacy hypothesis of masked semantic priming can be tested and confirmed. 

Support for a unitary account of masked priming effects also comes from influences of temporal uncertainty reduction on masked semantic priming and masked sensorimotor priming effects. Kiefer and Brendel (2006) used a warning signal for an upcoming masked priming word, and found that masked semantic priming was restricted to conditions with a relatively short interval between the warning signal and masked prime. Along similar lines, accessory stimuli that are used as a warning signal have a modulating effect on the amount of sensorimotor priming exerted by a masked arrow prime (Fischer, Schubert, & Liepelt, 2007).

Still, however, other findings, such as different time courses of masked semantic priming and masked response priming effects, point in the direction of differences between the underlying responsible faculties of unconscious vision. So far, however, any conclusions must be tentative, because the comparison between masked semantic and masked sensorimotor priming suffers from confounding differences between the stimuli or their relevant features. Most importantly, evidence for different time courses of masked semantic and masked response priming could be due to the use of mostly spatial and orientation information in masked sensorimotor priming studies, in contrast to the use of nonspatial meaning in masked semantic priming studies. With few exceptions (e.g., Klapp, 2005), studies of inverse masked priming used invisible location information of one or another kind.

Therefore, we suggest that masked semantic priming effects should be tested with words that have spatial meaning and thus bear a close resemblance to the typical features used in masked sensorimotor and attentional priming studies. For three related reasons, spatial meaning should be used for that purpose in future masked semantic priming studies. First, physical spatial information is responsible for many of the masked sensorimotor and attentional effects. The reason is obvious. Spatial information is shared by sensory and motor systems. It provides a common code across these domains, so to say (Prinz, 1990, 1997). Second and related, a large number of different effects have been detailed in masked sensorimotor priming studies by the use of masked spatial information. Examples are inversions of the priming effect, with better performance under incongruent than congruent conditions, once prime-target intervals exceed about 100 ms (cf. Eimer & Schlaghecken, 1998), or additive effects of spatial target-response correspondence/non-correspondence and spatial prime-target correspondence/noncorrespon-dence (Leuthold & Kopp, 1998). (Other examples were given above.) Third and finally, it was noted above that some evidence for an embodied cognition view of semantic processing was found in investigations of spatial word meaning (e.g., Proctor & Vu, 2002).

### Masked priming and theories of backward masking

 The majority of theories of backward masking focus more or less solely on the perception of the masked test stimulus (e.g., Kahneman, 1968; Stigler, 1910; Weisstein, 1968; for reviews see Breitmeyer, 1984, this volume; Breitmeyer & Öğmen, 2006), although some of these theories already contained less clearly stated implications for mask perception too (e.g., Bridgeman, 1971, this volume). Breitmeyer (1984), for instance, attributed the diminished visibility of the metacontrast masked test stimulus to inhibition exerted by fast transient channel activity (carrying information about mask onset) on activity in sustained channels (carrying information about test stimulus color and shape). An explanation of (diminished) test stimulus perception is also central to some recent mathematical models of backward masking (e.g., Breitmeyer & Öğmen, 2000; Francis, 1997; Francis & Herzog, 2004). 

A second class of backward masking theories additionally seeks to explain aspects of (conscious) mask perception (Bachmann, 1984, 1994; Di Lollo, Enns, & Rensink, 2000; Hamker, this volume; Herzog, Ernst, Etzold, & Eurich, 2003; Neumann, 1982; Neumann & Scharlau, in press; Scharlau, 2002). Herzog et al. (2003), for example, explain how features of the masked test stimulus can contribute to the phenomenal appearance of the mask’s shape (e.g., Herzog & Koch, 2001; Otto, Öğmen, & Herzog, 2006; Werner, 1935). Others (Bachmann, 1994; Neumann, 1982; Scharlau, 2002) gave accounts of temporal aspects of mask perception – that is, the decreased latency of perceiving a mask to a similar stimulus when it is not masking a preceding masked test stimulus.

A third class of models seeks to explain masked priming effects – that is, behavioral instead of perceptual effects (Vorberg et al., 2003). According to Vorberg and colleagues, the prime activates a response, and this activation accumulates for the duration that the masked prime is presented in isolation. Once the visible target commences, however, target-induced response activation kicks in that either adds to the already accumulated prime activity (because the visible target indicates the same response as the masked prime) or diminishes it (because the visible target indicates an alternative response). In both cases, a particular overt response will be executed, once a threshold of activity for that particular response has been passed. As a consequence, the execution of a particular response will occur fast after the onset of the visible target if masked prime and visible target activate one and the same response.

Finally, some theories account for both perceptual aspects of metacontrast masking and masked priming effects (Bowman, Schlaghecken, & Eimer, 2006; Lamme & Roelfsema, 2000). According to Lamme and Roelfsema, for instance, masked priming effects could be due to visual information being passed to successive visual and association cortex areas (hence, being available for response activation) during the first 100 ms after stimulus onset, a phase called the feedforward sweep by the authors. Conscious perception of a visual stimulus, however, would occur only during the following recurrent processing phase, during which initial stimulus-induced activity is confirmed by feedback activity from more anterior areas reaching back to the early visual cortical areas. As a consequence of this general architecture, a masked prime can activate a response (and is processed in some extent), despite the fact that a mask prevents visibility of the same prime stimulus because the mask prevents the confirmation of the feedforward signal triggered by the prime stimulus during the reentrant phase.

Bowman, Schlaghecken, and Eimer (2006), argue that reduced visibility of the masked prime stimulus is created by feedforward-driven competition between different possible perceptual states, and that inverted response priming effects are due to another mechanism, recurrent lateral inhibition between alternative response nodes.

Despite the diversity of these different approaches to explain backward masking and/or masked priming, all of the reviewed theories and models share the fundamental assumption that masked priming effects are inconsequential for what can be visually perceived in masking situations. Yet there are two good reasons to consider this possibility. First, according to the sensorimotor supremacy hypothesis, overt motor behavior provides building blocks for conscious perception (cf. Gurwitsch, 1964; O’Regan & Noë, 2001; Strauss, 1963). Thus, different latencies of sensorimotor processes could impact latencies of conscious perception. To our knowledge, there is so far only one model that acknowledges this possibility and has been suggested to apply to masked priming effects: According to the MMC (Mean of Multiple Computation) model, sensorimotor priming effects are due to independent feedforward inputs by (a) the masked prime and (b) the visible target, whereas conscious perception of the visible target corresponds to a stable attractor state of the artificial neural net that is only achieved after several iterations of forward and backward propagated activity (Cruse, 2003). It should be noted that the model was originally developed to give an account of pointing directions. At present, it admittedly awaits its detailed application to the results of masked priming studies.

Crucially in the current context, however, the MMC model predicts that the neural net achieves its stable attractor state faster under conditions in which masked prime and visible target activate similar responses than under conditions in which masked prime and visible target activate alternative responses, respectively. So far, this prediction of the MMC model seems not to be supported by the evidence. Scharlau and Ansorge (2003), for instance, found similar amounts of perceptual latency facilitation under both the aforementioned conditions. Yet, under all of these conditions, masked primes also allowed for attentional facilitation. Therefore, attentional facilitation as a common effect of both response-congruent and response-incongruent primes could have occurred instead of a perceptual latency inhibition by the sensorimotor effects of the incongruent masked primes (relative to the congruent primes) in studies such as that of Scharlau and Ansorge. Future studies should prevent attentional facilitation by the masked prime from occuring instead of sensorimotor processing of the masked primes, for example, by presenting all stimuli at already attended-to locations, so that common attentional effects of the primes are undermined.

A second implication of masked priming research for backward masking theories is that goal settings impact on sensorimotor processing of masked visual stimuli (cf. Ansorge & Neumann, 2005; Kunde et al., 2003) in general and the distribution of attention to masked stimuli in particular (Ansorge & Neumann, 2005; Scharlau & Ansorge, 2003). The likely reason for this is that the extent of a match between a goal setting and a feature of a masked stimulus changes the visual processing dynamics: There is evidence from research with visible stimuli that a goal setting (or working memory content) can determine both (a) the latency with which attention can be directed to a particular visual feature (cf. Ansorge & Horstmann, 2007; Ansorge, Horstmann, & Carbone, 2005; Soto, Heinke, Humphreys, & Blanco, 2005) and (b) the duration with which attention is kept on a particular visual stimulus (Eriksen & Yeh, 1985; Theeuwes, Atchley, & Kramer, 2000). With a visible stimulus that matches the goal settings (say a red stimulus if observers search for something red), the latency with which attention can be directed to that stimulus is curtailed and the duration with which attention is kept on that stimulus is prolonged. Moreover, at least the latter seems to hold true for unconscious visual stimuli too (Ivanoff & Klein, 2003).

Now because (a) goal settings determine where humans direct their attention and (b) the direction and distribution of attention to a stimulus is necessary for conscious visual perception of the same stimulus and thus precedes it (cf. Neisser, 1967; Treisman & Gelade, 1980), we should expect collateral effects of goal settings set up for sensorimotor processing purposes on the latency of conscious visual stimulus perception too, in line with the observation that the extent of a match between a goal setting and a masked visual stimulus directly impacts on the latency of conscious mask perception (Scharlau & Ansorge, 2003). There are several models and theories of visual attention that could in principle be applied to detail the corresponding influences in backward masking and masked priming theories (e.g., Desimone & Duncan, 1995; Hamker, 2004; VanRullen & Thorpe, 1999). However, again, these are conceivable applications of the models which have to await future research.

### Summary

The current report showed that the sensorimotor supremacy hypothesis is both well supported by a large body of evidence and rich in new predictions for future research. In the first part of our report, we reviewed different kinds of masked priming effects, sensorimotor, attentional, and semantic priming. We argued that masked sensorimotor priming is very good evidence for the sensorimotor supremacy hypothesis: According to this hypothesis, conscious perception draws on motor behavior, and thus follows sensorimotor processing, and therefore can be disrupted at a point in time by a backward mask at which response-activation effects already escaped the influence of the mask. We also suggested that masked attentional and semantic priming effects could reflect variants of sensorimotor priming – that is, premotor specification of motor parameters and partial re-instantiations of prior sensorimotor processes in memory, respectively. Finally, we ended the first part of our report by suggesting ways to test the sensorimotor account of masked attentional and masked semantic priming effects.

In the second part of our report, we reviewed different theories and models of backward masking and masked priming effects, and concluded that these do not fully acknowledge possible bearings that masked priming effects have on any theory of backward masking. We proceeded by detailing two of these bearings from masked priming research, impacts that (a) sensorimotor processes and (b) goal settings can have on what is perceived and at what time under backward masking conditions. Finally, we summarized some of the existing motor and attention theories and models which could be used in future research to account for the so far unacknowledged bearings of masked priming effects on backward masking.

## References

[R1] Allport A., Heuer H., Sanders A. F. (1987). Selection for action: Some behavioral and neurophysiological
						considerations of attention and action.. Perspectives on perception and action.

[R2] Ansorge U. (2003). Asymmetric influences of temporally vs. nasally presented masked
						visual information: Evidence for collicular contributions to nonconscious
						priming effects.. Brain and Cognition.

[R3] Ansorge U. (2004). Top-down contingencies of nonconscious priming revealed by
						dual-task interference.. Quarterly Journal of Experimental Psychology.

[R4] Ansorge U., Heumann M. (2003). Top-down contingencies in peripheral cuing: The roles of color
						and location.. Journal of Experimental Psychology: Human Perception and
						Performance.

[R5] Ansorge U., Heumann M. (2004). Peripheral cuing by abrupt-onset cues: The role of color in S-R
						corresponding conditions.. Acta Psychologica.

[R6] Ansorge U., Heumann M. (2006). Shifts of visuospatial attention to invisible
						(metacontrast-masked) singletons: Clues from reaction times and
						event-related potentials.. Advances in Cognitive Psychology.

[R7] Ansorge U., Heumann M., Scharlau I. (2002). Influences of visibility, intentions, and probability in a
						peripheral cuing task.. Consciousness and Cognition.

[R8] Ansorge U., Horstmann G. (2007). Preemptive control of attentional capture by color: Evidence from
						trial-by-trial analysis and ordering of onsets of capture effects in RT
						distributions.. Quarterly Journal of Experimental Psychology.

[R9] Ansorge U., Horstmann G., Carbone E. (2005). Top-down contingent capture by color: Evidence from RT
						distribution analyses in a manual choice reaction task.. Acta Psychologica.

[R10] Ansorge U., Klotz W., Neumann O. (1998). Manual and verbal responses to completely masked (unreportable)
						stimuli: Exploring some conditions for the metacontrast
						dissociation.. Perception.

[R11] Ansorge U., Neumann O. (2001). Intentions determine the effect of nonconsciously registered
						visual information: Evidence for direct parameter specification in the
						metacontrast dissociation..

[R12] Ansorge U., Neumann O. (2005). Intentions determine the effect of invisible metacontrast-masked
						primes: Evidence for top-down contingencies in a peripheral cueing
						task.. Journal of Experimental Psychology: Human Perception and
						Performance.

[R13] Bachmann T. (1984). The process of perceptual retouch: Nonspecific afferent
						activation dyna-mics in explaining visual masking.. Perception & Psychophysics.

[R14] Bachmann T. (1994). Psychophysiology of visual masking: The fine structure of conscious
						experience..

[R15] Barsalou L. W. (1999). Perceptual symbol systems.. Behavioral and Brain Sciences.

[R16] Bowman H., Schlaghecken F., Eimer M. (2006). A neural network model of inhibitory processes in subliminal
						priming.. Visual Cognition.

[R17] Breitmeyer B. G. (1984). Visual masking: An integrative approach..

[R17a] Breitmeyer B. G. (2007). Visual Masking: Past accom-plishments, present status, future
						developments.. Advances in Cognitive Psychology.

[R18] Breitmeyer B. G., Öğmen H. (2000). Recent models and findings in visual backward masking: A
						comparison, review, and update.. Perception & Psychophysics.

[R19] Breitmeyer B. G., Öğmen H. (2006). Visual masking: Time slices through conscious and unconscious
						vision..

[R20] Breitmeyer B. G., Ro T., Singhal N. S. (2004). Unconscious color priming occurs at stimulus- not
						percept-dependent levels of processing.. Psychological Science.

[R21] Bridgeman B. (1971). Metacontrast and lateral inhibition.. Psychological Review.

[R22] Bridgeman B. (1992). Conscious vs. unconscious processes. The case of
						vision.. Theory and Psychology.

[R22a] Bridgeman B. (2007). Common-onset masking simulated with a distributed-code
						model.. Advances in Cognitive Psychology.

[R23] Bridgeman B., Lewis S., Heit G., Nagle M. (1979). Relation between cognitive and motor-oriented systems of visual
						position perception.. Journal of Experimental Psychology: Human Perception and
						Performance.

[R24] Cheesman J., Merikle P. M., Besner D., Waller T. G., MacKinnon G. E. (1985). Word recognition and consciousness.. Reading research: Advances in theory and practice.

[R25] Cruse H. (2003). The evolution of cognition – a
						hypothesis.. Cognitive Science.

[R26] Dehaene S., Naccache L. (2001). Towards a cognitive neuroscience of consciousness: Basic evidence
						and a workspace framework.. Cognition.

[R27] Dehaene S., Naccache L., Le Clec’H G., Koechlin E., Mueller M., Dehaene-Lambertz G., van de Moortele P., Le Bihan D. (1998). Imaging unconscious semantic priming.. Nature.

[R28] Desimone R., Duncan J. (1995). Neural mechanisms of selective visual attention.. Annual Review of Neuroscience.

[R29] Deubel H., Schneider W. X. (1996). Saccade target selection and object recognition: Evidence for a
						common attentional mechanism.. Vision Research.

[R30] Deubel H., Schneider W. X., Humphreys G. W., Riddoch M. J. (2004). Attentional selection in sequential manual movements, movements
						around an obstacle, and in grasping.. Attention in action.

[R31] Di Lollo V., Enns J. T., Rensink R. A. (2000). Competition for consciousness among visual events: The
						psychophysics of reentrant visual processes.. Journal of Experimental Psychology: General.

[R32] Dimberg U., Thunberg M., Elmehed K. (2000). Unconscious facial reactions to emotional facial
						expressions.. Psychological Science.

[R33] Eckstein D., Perrig W. J. (2007). The influence of intention on masked priming: A study with
						semantic classification of words.. Cognition.

[R34] Eimer M. (1999). Facilitatory and inhibitory effects of masked prime stimuli on
						motor activation and behavioural performance.. Acta Psychologica.

[R35] Eimer M., Schlaghecken F. (1998). Effects of masked stimuli on motor activation: Behavioral and
						electrophysiological evidence.. Journal of Experimental Psychology: Human Perception and
						Performance.

[R36] Eimer M., Schlaghecken F. (2001). Response facilitation and inhibition in manual, vocal, and
						oculomotor performance: Evidence for a modality-unspecific
						mechanism.. Journal of Motor Behaviour.

[R37] Enns J. T., Di Lollo V. (2000). What’s new in visual masking?. Trends in Cognitive Sciences.

[R38] Eriksen C. W., Yeh Y-Y. (1985). Allocation of attention in the visual field.. Journal of Experimental Psychology: Human Perception and
						Performance.

[R39] Fehrer E., Raab D. (1962). Reaction time to stimuli masked by metacontrast.. Journal of Experimental Psychology.

[R40] Fischer R., Schubert T., Liepelt R. (2007). Accessory stimuli modulate effects of non-conscious
						priming.. Perception & Psychophysics.

[R41] Folk C. L., Remington R. W. (1998). Selectivity in distraction by irrelevant featural singletons:
						Evidence for two forms of attentional capture.. Journal of Experimental Psychology: Human Perception and
						Performance.

[R42] Folk C. L., Remington R. W. (1999). Can new objects override attentional control
						settings?. Perception & Psychophysics.

[R43] Folk C. L., Remington R. W., Johnston J. C. (1992). Involuntary covert orienting is contingent on attentional control
						settings.. Journal of Experimental Psychology: Human Perception and
						Performance.

[R44] Francis G. (1997). Cortical dynamics of lateral inhibition: Metacontrast
						masking.. Psychological Review.

[R45] Francis G., Herzog M. (2004). Testing quantitative models of backward masking.. Psychonomic Bulletin & Review.

[R46] Goodale M. A., Milner A. D., Jakobson L. S., Carey D. P. (1991). A neurological dissociation between perceiving objects and
						grasping them.. Nature.

[R47] Goodale M. A., Milner A. D. (1992). Separate visual pathways for perception and
						action.. Trends in Neurosciences.

[R48] Greenwald A. G., Draine S. C., Abrams R. L. (1996). Three cognitive markers of unconscious semantic
						activation.. Science.

[R49] Gurwitsch A. (1964). The field of consciousness..

[R50] Hamker F. H. (2004). A dynamic model of how feature cues guide spatial
						attention.. Vision Research.

[R51] Hamker F. H. (2007). The mechanisms of feature inheritance as predicted by a
						systems-level model of visual attention and decision making.. Advances in Cognitive Psychology.

[R52] Hardcastle V. G. (1995). Locating consciousness..

[R53] Helmholtz H. v. (1879). Die Thatsachen in der Wahrnehmung.

[R54] Herzog M. H., Ernst U., Etzold A., Eurich C. (2003). Local interactions in neural networks explain global effects in
						the masking of visual stimuli.. Neural Computation.

[R55] Herzog M. H., Koch C. (2001). Seeing properties of an invisible object: Feature inheritance and
						shine-through.. Proceedings of the National Academy for Science, USA.

[R56] Hoffmann J. (1993). Vorhersage und Erkenntnis..

[R57] Ivanoff J., Klein R. M. (2003). Orienting of attention without awareness is affected by
						measurement-induced attentional control settings.. Journal of Vision.

[R58] James W. (1890). The principles of psychology..

[R59] Jaśkowski P., Skalska B., Verleger R. (2003). How the self controls its “automatic pilot”
						when processing subliminal information.. Journal of Cognitive Neuroscience.

[R60] Jaśkowski P., Verleger R. (2007). What determines the direction of subliminal
						priming.. Advances in Cognitive Psychology.

[R61] Jonides J., Long J. B., Baddeley A. D. (1981). Voluntary versus automatic control over the mind’s
						eye’s movement.. Attention and performance IX.

[R62] Kahneman D. (1968). Method, findings, and theory in studies of visual
						masking.. Psychological Bulletin.

[R63] Kiefer M. (2002). The N400 is modulated by unconsciously perceived masked words:
						Further evidence for a spreading activation account of N400 priming
						effects.. Cognitive Brain Research.

[R64] Kiefer M. (2007). Top-down modulation of unconscious
						‘automatic’ processes: A gating
						framework.. Advances in Cognitive Psychology.

[R65] Kiefer M., Brendel D. (2006). Attentional modulation of unconscious semantic processes:
						Evidence from event-related potentials in masked priming
						paradigm.. Journal of Cognitive Neuroscience.

[R66] Kiefer M., Spitzer M. (2000). Time course of conscious and unconscious semantic brain
						activation.. NeuroReport.

[R67] Kiesel A., Kunde W., Hoffmann J. (2007). Mechanisms of subliminal response priming.. Advances in Cognitive Psychology.

[R68] Klapp S. T. (2005). Two versions of the negative compatibility effect: A reply to
						LLeras and Enns (2004).. Journal of Experimental Psychology: General.

[R69] Klapp S. T., Hinkley L. B. (2002). The negative compatibility effect: Unconscious inhibition
						influences reaction time and response selection.. Journal of Experimental Psychology: General.

[R70] Klotz W., Neumann O. (1999). Motor activation without conscious discrimination in metacontrast
						masking.. Journal of Experimental Psychology: Human Perception and
						Performance.

[R71] Klotz W., Wolff P. (1995). The effect of a masked stimulus on the response to the masking
						stimulus.. Psychological Research.

[R72] Kunde W., Kiesel A., Hoffmann J. (2003). Conscious control over the content of unconscious
						cognition.. Cognition.

[R73] Kutas M., Hillyard S. A. (1980). Reading senseless sentences – brain potentials reflect
						semantic incongruity.. Science.

[R74] Lamme V. A., Roelfsema P. R. (2000). The distinct modes of vision offered by feedforward and recurrent
						processing.. Trends in Cognitive Neurosciences.

[R75] Leuthold H., Jentzsch I. (2002). Distinguishing neural sources of movement preparation and
						execution: An electrophysiological analysis.. Biological Psychology.

[R76] Leuthold H., Kopp B. (1998). Mechanisms of priming by masked stimuli: Inferences from
						event-related brain potentials.. Psychological Science.

[R77] Lleras A., Enns J. T. (2004). Negative compatibility or object updating? A cautionary tale of
						mask-dependent priming.. Journal of Experimental Psychology: General.

[R78] Lleras A., Enns J. T. (2005). Updating a cautionary tale of masked priming: A reply to Klapp
						(2005).. Journal of Experimental Psychology: General.

[R79] Lleras A., Enns J. T. (2006). How much like a target can a mask be? Geometric, spatial, and
						temporal similarity in priming – A reply to Schlaghecken and
						Eimer.. Journal of Experimental Psychology: General.

[R80] Lu C. H., Proctor R. W. (1995). The influence of irrelevant location information on performance:
						A review of the Simon and spatial Stroop effects.. Psychonomic Bulletin & Review.

[R81] Marcel A. J. (1983). Conscious and unconscious perception: Experiments on visual
						masking and word recognition.. Cognitive Psychology.

[R82] McClelland J. L., Rumelhart D. E. (1981). An interactive activation model of context effects in letter
						perception: Part 1. An account of basic findings.. Psychological Review.

[R83] McCormick P. A. (1997). Orienting attention without awareness.. Journal of Experimental Psychology: Human Perception and
						Performance.

[R84] Morton J. (1969). The interaction of information in word
						recognition.. Psychological Review.

[R85] Neely J. H. (1977). Semantic priming and retrieval from lexical memory: Roles of
						inhibitionless spreading of activation and limited-capacity
						attention.. Journal of Experimental Psychology: General.

[R86] Neely J. H., Besner D., Humphreys G. W. (1991). Semantic priming effects in visual word recognition: A selective
						review of current findings and theories.. Basic processes in reading: Visual word recognition.

[R87] Neisser U. (1967). Cognitive Psychology..

[R88] Neumann O. (1982). Experimente zum Fehrer-Raab-Effekt und das
						‘Wetterwart’-Modell der visuellen
						Maskierung.

[R89] Neumann O., Heuer H., Sanders A. F. (1987). Beyond capacity: A functional view of attention.. Perspectives on perception and action.

[R90] Neumann O. (1989). Kognitive Vermittlung und direkte Para-meter-spezifikation. Zum
						Problem mentaler Repräsentation in der Wahrnehmung.. Sprache und Kognition.

[R91] Neumann O. (1990). Direct parameter specification and the concept of
						perception.. Psychological Research/Psychologische Forschung.

[R92] Neumann O., Klotz W., Umiltà C., Moscovitch M. (1994). Motor responses to nonreportable, masked stimuli: Where is the
						limit of direct parameter specification?. Attention and performance XV: Conscious and nonconscious information
						processing.

[R93] Neumann O., Scharlau I. Experiments on the Fehrer-Raab effect and the
						‘Wheather Station Model’ of visual backward
						masking.. Psychological Research.

[R94] Newell A., Simon H. (1972). Human problem solving..

[R95] Nijhawan R. (2002). Neural delays, visual motion and the flash-lag
						effect.. Trends in Cognitive Sciences.

[R96] O’Regan J. K., Noë A. (2001). A sensorimotor account of vision and visual
						consciousness.. Behavioral and Brain Sciences.

[R97] Otto T. U., Öğmen H., Herzog M. H. (2006). The flight of the Phoenix – the visible trace of
						invisible elements in human vision.. Journal of Vision.

[R98] Posner M. I., Snyder C. R. R., Solso R. L. (1975). Attention and cognitive control.. Information processing and cognition.

[R99] Prinz W., Neumann O., Prinz W. (1990). A common coding approach to perception and
						action.. Relationships between perception and action.

[R100] Prinz W. (1997). Perception and action planning.. European Journal of Cognitive Psychology.

[R101] Proctor R. W., Vu K.-P. L. (2002). Mixing incompatibly mapped location-irrelevant trials and
						location-relevant trials: Influence of stimulus mode on spatial
						compatibility effects.. Memory & Cognition.

[R102] Pylyshyn Z. (1984). Computation and cognition..

[R103] Reynvoet B., Gevers W., Caessens B. (2005). Unconscious primes activate motor codes through
						semantics.. Journal of Experimental Psychology: Learning, Memory, and
						Cognition.

[R104] Riggs L. A., Ratliff F., Cornsweet J. C., Cornsweet T. N. (1953). The disappearance of steadily fixated visual test
						objects.. Journal of the Optical Society of America.

[R105] Rizzolatti G., Riggio L., Dascola I., Umiltà C. (1987). Reorienting attention across the horizontal and vertical
						meridians: Evidence in favor of a premotor theory of
						attention.. Neuropsychologia.

[R106] Rizzolatti G., Riggio L., Sheliga B. M., Umiltà C., Moscovitch M. (1994). Space and selective attention.. Attention and Performance, XV: Conscious and Nonconscious Information
						Processing.

[R107] Scharlau I. (2002). Leading, but not trailing, primes influence temporal order
						perception: Further evidence for an attentional account of perceptual
						latency priming.. Perception & Psychophysics.

[R108] Scharlau I. (2007). Temporal processes in prime-mask interaction: Assessing
						perceptual consequences of masked information.. Advances in Cognitive Psychology.

[R109] Scharlau I., Ansorge U. (2003). Direct parameter specification of an attention shift: Evidence
						from perceptual latency priming.. Vision Research.

[R110] Scharlau I., Ansorge U., Horstmann G. (2006). Latency facilitation in temporal-order judgments: Time course of
						facilitation as a function of judgment type.. Acta Psychologica.

[R111] Scharlau I., Neumann O. (2003a). Perceptual latency priming by masked and unmasked stimuli:
						Evidence for an attentional interpretation.. Psychological Research/Psychologische Forschung.

[R112] Scharlau I., Neumann O. (2003b). Temporal parameters and time course of perceptual latency
						priming.. Acta Psychologica.

[R113] Schlaghecken F., Eimer M. (2002). Motor activation with and without inhibition: Evidence for a
						threshold mechanism in motor control.. Perception & Psychophysics.

[R114] Schlaghecken F., Eimer M. (2004). Masked stimuli can bias “free” choices
						between response alternatives.. Psychonomic Bulletin & Review.

[R115] Schlaghecken F., Rowley L., Sembi S., Simmons R., Whitcomb D. (2007). The negative compatibility effect: A case for
						self-inhibition.. Advances in Cognitive Psychology.

[R116] Schmidt T., Metzinger T. (2001). Visual perception without awareness: Priming responses by
						color.. Neural correlates of consciousness.

[R117] Schmidt T. (2002). The finger in flight: Real-time motor control by visually masked
						color stimuli.. Psychological Science.

[R118] Schmidt T. (2007). Measuring unconscious cognition: Beyond the zero-awareness
						criterion.. Advances in Cognitive Psychology.

[R119] Schmidt T., Niehaus S., Nagel A. (2006). Primes and targets in rapid chases: Tracing sequential waves of
						motor activation.. Behavioral Neuroscience.

[R120] Simons D. J., Rensink R. A. (2005). Change blindness: Past, present, and future.. Trends in Cognitive Sciences.

[R121] Soto D., Heinke D., Humphreys G. W., Blanco M. J. (2005). Early, involuntary top-down guidance of attention from working
						memory.. Journal of Experimental Psychology: Human Perception, and
						Performance.

[R122] Sumner P. (2007). Negative and positive masked priming – implications
						for motor inhibition.. Advances in Cognitive Psychology.

[R123] Stigler R. (1910). Chronoptische Studien über den Umgebungskontrast
						[Chronoptical studies on the
						„Umgebungskontrast“].. Pflüger’s Archiv für die gesamte
						Physiologie.

[R124] Strauss E. (1963). The primary world of senses..

[R125] Theeuwes J., Atchley P., Kramer A. F., Monsell S., Driver J. (2000). On the time course of top-down and bottom-up control of visual
						attention.. Attention and performance XVIII.

[R126] Treisman A. M., Gelade G. (1980). A feature-integration theory of attention.. Cognitive Psychology.

[R127] VanRullen R., Thorpe S. J. (1999). Spatial attention in asynchronous neural
						networks.. Neurocomputing.

[R128] Verleger R., Jaśkowski P., Aydemir A., Van der Lubbe R. H. J., Groen M. (2004). Qualitative differences between conscious and non-conscious
						processing? On negative and positive priming effects induced by masked
						arrows.. Journal of Experimental Psychology: General.

[R129] Vorberg D., Mattler U., Heinecke A., Schmidt T., Schwarzbach J. (2003). Different time courses for visual perception and action
						priming.. Proceedings of the National Academy of Science.

[R130] Weisstein N. (1968). A Rashevsky-Landahl neural net: Simulation of
						metcontrast.. Psychological Review.

[R131] Werner H. (1935). Studies on contour: I. Qualitative analyses.. American Journal of Psychology.

[R132] Wilson M. (2002). Six views of embodied cognition.. Psychonomic Bulletin & Review.

[R132a] Wolff P. (1989). Einfluß des maskierten Testreizes auf die
						Wahlreaktion auf den Metakontrast. Visual Cognition.

[R133] Wolff P. (2004). Position of code and code for position: From isomorphism to a
						sensorimotor account of space perception.. Visual Cognition.

[R134] Woodman G. F., Luck S. J. (2003). Dissociations among attention, perception, and awareness during
						object-substitution masking.. Psychological Science.

[R135] Yantis S., Jonides J. (1984). Abrupt visual onsets and selective attention: Evidence from
						visual search.. Journal of Experimental Psychology: Human Perception and
						Performance.

